# Care for Critical Ill Patients With COVID-19: Establishment of a Temporary Intensive Care Unit in an Isolated Hospital

**DOI:** 10.3389/fmed.2020.00519

**Published:** 2020-08-11

**Authors:** Milin Peng, Zhaoxin Qian, Lina Zhang

**Affiliations:** ^1^Department of Critical Care Medicine, Xiangya Hospital, Central South University, Changsha, China; ^2^National Clinical Research Center for Geriatric Disorders, Xiangya Hospital, Central South University, Changsha, China; ^3^Department of Cardiovascular, Xiangya Hospital, Central South University, Changsha, China

**Keywords:** COVID-19, management, severe, temporary, intensive care unit

## Abstract

The current global spread of COVID-19, a highly contagious disease, has challenged healthcare systems, and placed immense burdens on medical staff globally. With a sharp increase in the number of newly confirmed cases and the rapid progression of the disease into a critically ill state, overstretched critical care units have had to contend with a shortage of beds, specialist personnel, and medical resources. Temporary intensive care units (ICUs) were therefore set up in isolated hospitals to provide the required standardized care for all severe cases. The current paper describes the authors' experience of setting up and managing such an ICU in Wuhan, Hubei Province, China, from the identification of critically ill COVID-19 patients through to the arranging and equipping of the unit, providing training and protection for staff, and standardizing all aspects of care.

## Introduction

Coronavirus Disease 2019 (COVID-19), caused by severe acute respiratory syndrome coronavirus 2 (SARS-CoV-2), is highly contagious. In recognition of the global threat it poses, on March 11, 2020, the World Health Organization (WHO) declared COVID-19 to be a pandemic. By July 22, 2020, the total officially confirmed cases in China reached 86,152 with 4,653(5.4%) having died since the outbreak began in December 2019 (https://covid19.who.int/). COVID-19 has high mortality throughout the world, being especially high in Italy (*n* = 236,076, mortality with 32,867/236,076), Spain (*n* = 264,836, mortality with 28,422/264,836), Russia (*n* = 783,328, mortality with 12,580/783,328), Brazil (*n* = 2,098,389, mortality with 79,488/2,098,389), the United States (*n* = 3,748,248, mortality with 139,964/3,748,248), and South Africa (*n* = 373,628, mortality with 5,173/373,628) (https://covid19.who.int/, https://www.epicentro.iss.it/en/coronavirus/bollettino/Infografica_10giugno%20ENG.pdf). The high incidence and mortality of COVID-19 puts pressure on the need for urgent and special requirements for global medical resources and infrastructures.

## Purpose and Significance of a Temporary COVID-19 ICU

Data provided by the Chinese Center for Disease Control and Prevention revealed a total of 44,672 confirmed COVID-19 cases in China by February 11, 2020. Of these, the majority (87%) were aged between 30 and 79, while 3% were aged 80 or more. A total of 14% of these patients were, or had been, severely ill, and 5% required critical care (1). Data from a single-center, retrospective study of 52 critically ill COVID-19 patients showed that 61.5% died at 28 days, 71% required mechanical ventilation, and 67% had acute respiratory distress syndrome (ARDS). Patients who died from the disease were recorded longer lingering time from onset of symptoms to ICU admission than those who survived (2).

As the number of newly-confirmed and critical COVID-19 cases rose sharply from December 2019 onwards in Wuhan, isolated hospitals charged with caring for such patients had to solve immense challenges in terms of finding sufficient bed space, personnel, and resources, particularly in relation to the provision of care for the critically ill patients (3). As COVID-19 is characterized by a high rate of contagion and rapid progression coupled with a high mortality rate, especially in the case of critical patients, it was essential to identify potentially critical cases at the earliest possible stage so they could be transferred to ICU timely to achieve necessary respiratory or circulation support in order to reduce mortality rates (4).

Given the shortage of critical care facilities and resources in isolated hospitals (5), the establishment of temporary ICUs in such hospitals was an essential part of the infrastructure for providing care to critically ill COVID-19 patients. The government thus made the decision to allocate an estimated 2,500 beds in three general hospitals in the city of Wuhan as temporary isolated wards for the treatment of severely infected patients. Over 3,000 medical personnel, including up to 1,000 ICU doctors and nurses, were called together from elsewhere in China to form centralized, specialist rescue teams in each isolated hospital. Each member of the teams represented a different department; thus, uniform and normative team management and patient care were challenging. Existing medical resources were leveraged to meet the needs of these ICUs, and the training of non-ICU staff was also critically important.

Over the course of a month in Wuhan, after the temporary ICU was established, a total of 157 critically ill patients were admitted into our temporary ward; they had characteristics of a mean age at 62 years old, a mean hospital stay at 16.01 days, 14.0% requiring invasive or non-invasive mechanical ventilation, and 3.82% died at discharge. Hence, the current paper shares best practices gleaned from the establishment and management of temporary ICUs in isolated hospitals during the epidemic in Wuhan; the centralization of severe confirmed cases enabled reintegration and maximum use of existing medical resources, thus facilitating effective professional treatments for COVID-19 patients.

## Establishment of a Temporary COVID-19 ICU

On February 7, 2020, a national medical team from Xiangya Hospital, Hunan, took over the 51-bed ophthalmic ward in the Union Hospital Tongji Medical College Huazhong University of Science and Technology, Wuhan, in response to the insufficient number of ICU beds in isolated hospitals to cope with the rapidly increasing numbers of cases presenting with COVID-19. Combining the characteristics of the ward and taking into consideration severe cases, we planned to establish a temporary ICU in the Ophthalmic Ward; however, given its previous use, not all beds were supplied with sufficient oxygen pressure to run the necessary ventilators.

### Early Identification and Sorting of Critical Cases

**Figure 1:**
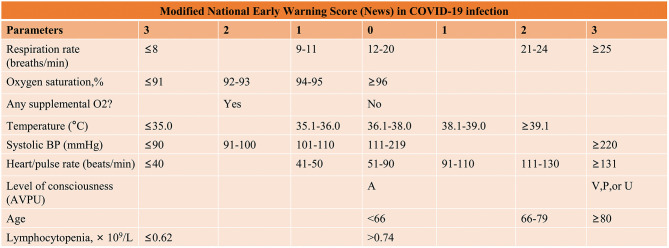
Early classification of critically COVID-19 patients. BP, blood pressure, level of consciousness; A, Alert; V, Responds to voice; P, Responds to pain; U, Unresponsive.

The first stage in the care of COVID-19 patients is to identify those who are critically ill. For this purpose, we modified the National Early Warning Score (News) (6) and SOFA score and added two further predicted risk factors for severe COVID-19 infection: age ≥ 66 years old (2, 7, 8) and persistent lymphocytopenia (2, 8, 9) (Figure 1). Post-admission, critically ill patients were sorted into one of four risk categories on the basis of their scores on the severity grading scales: mild, moderate, critical, and severe critical. It was important to recognize that critically ill patients who presented with silent hypoxemia (severe hypoxemia without signs of respiratory distress), particularly older ones, required vigilant monitoring. Initially, due to the lack of severe respiratory distress symptoms at the early stage of COVID-19 in some severe confirmed patients, clinicians might delay tracheal intubation, and invasive ventilation. This postponing would bring disastrous consequences for these patients. Hence, early identification of severe critical patients was crucial in enabling personnel to offer optimized treatment within the temporary ICU in the shortest time.

### Dividing the Ward Into Sectors

**Figure 2:**
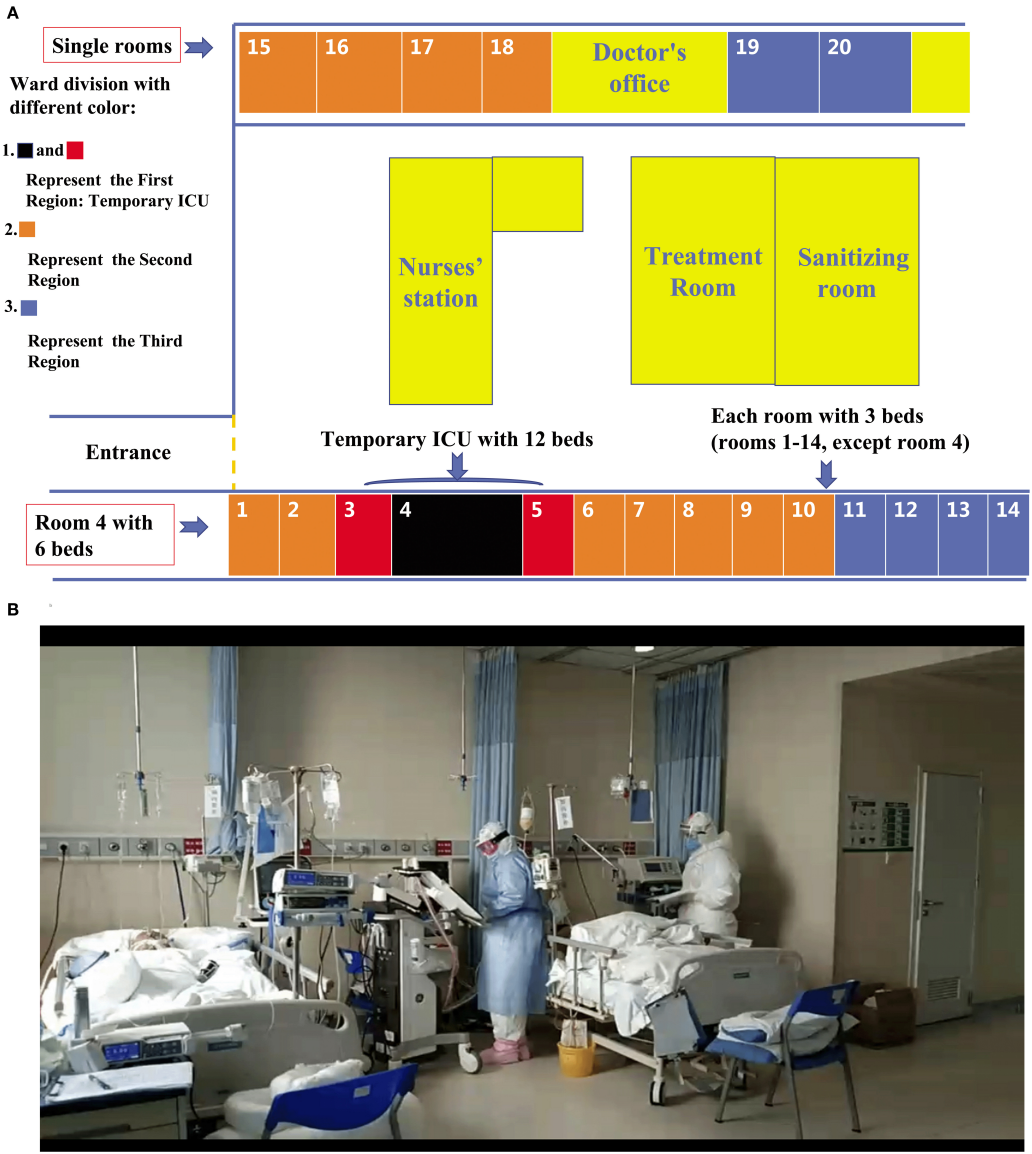
Dividing a ward into sectors. **(A)** After the ophthalmology ward was transformed into a temporary ICU, beds were arranged as follows: the first sector, consisting of 12 beds, made up the temporary ICU (room four with six beds; room three with three beds; and room five with three beds); the rooms shown in orange indicate the second sector, comprising 25 beds; the rooms shown in blue indicate the third sector, comprising 14 beds. **(B)** A team led by specialized ICU physicians viewing critically ill COVID-19 patients in a temporary ICU.

**Figure 3:**
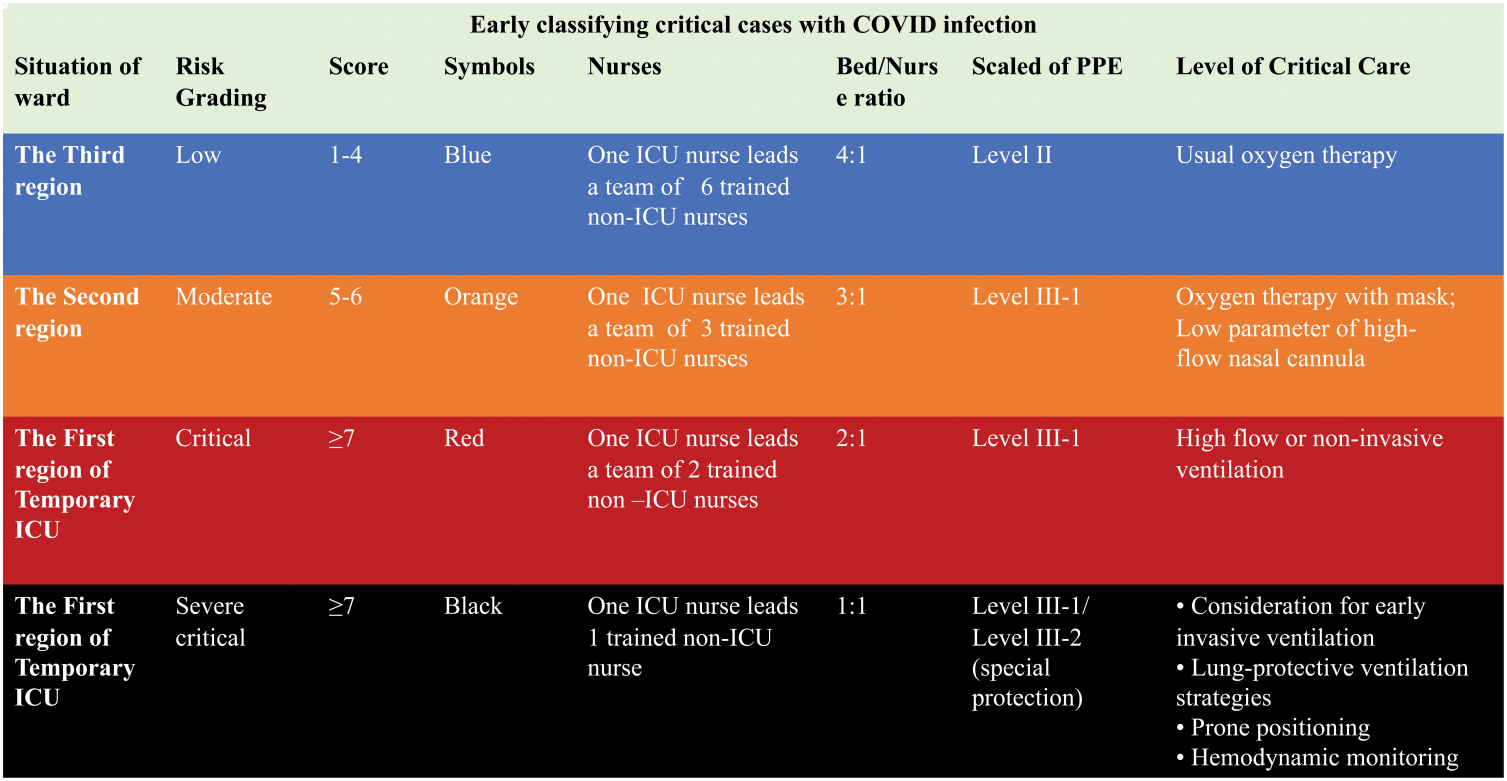
Early sorting of critically ill COVID-19 patients according to a modified Early Warning Score in a temporary ICU.

The ward was divided into three sectors according to the results of the severity grading scales to facilitate clinical management (Figures 2, 3).

**Figure 4:**
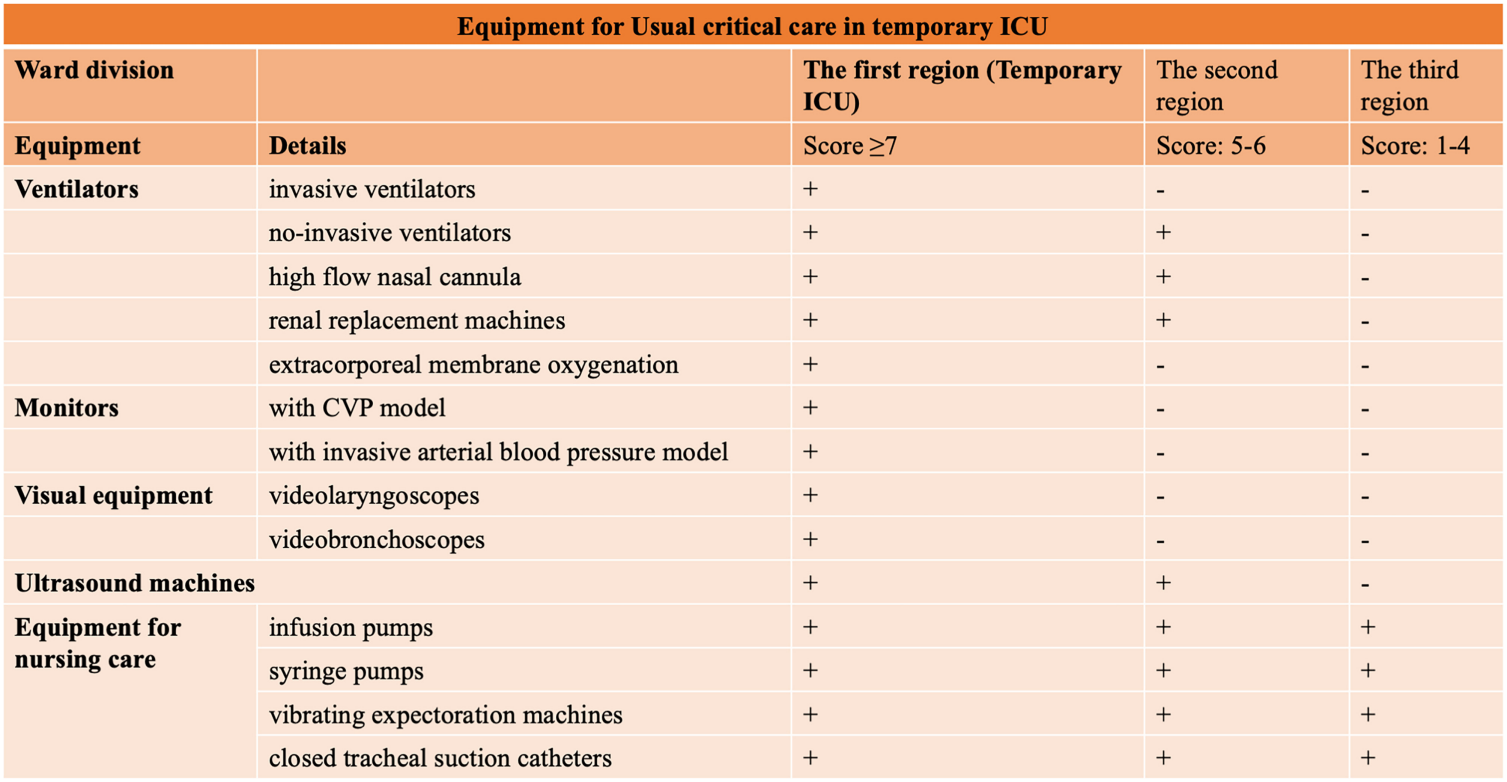
Summary of the requirements for special medical equipment in a temporary ICU caring for critically ill COVID-19 patients.

The first sector, containing 12 beds supplied with sufficient oxygen pressure to run ventilators, was designated for the management of severe critically ill patients, including those requiring invasive ventilator support or prone ventilation, exhibiting hemodynamic instability. This sector was located close to the nurses' and doctors' workstations, enabling staff to monitor patients closely and provide immediate attention. The beds were placed at least two meters apart to prevent cross-infection. Figure 4 displays the special medical equipment required in the temporary ICU, including non-invasive and invasive ventilators, high-flow nasal cannula (HFNC), renal replacement machines, extracorporeal membrane oxygenation machines, videobronchoscopes (with external monitor), equipment to monitor central venous pressure (CVP) or invasive arterial blood pressure, ultrasound machines, videolaryngoscopes, infusion or syringe pumps, vibrating expectoration machines, and closed tracheal suction catheters.

The second sector of the temporary ICU consisted of a ring of 25 beds outside the critical care sector and was designated for the care of moderate to critical patients, typically requiring low-parameter high flow oxygen therapy with a maximum flow rate of 40 L/min and inspired oxygen (FiO2) of 50%, or mask oxygen support with a maximum flow of 5 L/min. The third part, which consisted of 14 beds around the perimeter of the ward, was for the use of patients identified as moderate, or who were in the recovery stage or requiring nasal catheter oxygen.

### COVID-19 Infection Special Intensive Care Teams Establish

With insufficient trained ICU personnel to meet demand, we set up a multidisciplinary intensive care team led by senior intensive care specialists, and an intensive nurse team led by experienced intensive care nurses. Our first task was to evaluate isolation conditions, after which we divided the ward into inner and outer zones to give workers a space in which they could rest safely. Since every patient under our care was suffering from the same disease, we then took steps to standardize treatments by establishing protection strategy for each healthcare operation, for example routine medical care, tracheal intubation, and percutaneous tracheotomy.

### Protecting Patients and Health Workers

**Figure 5:**
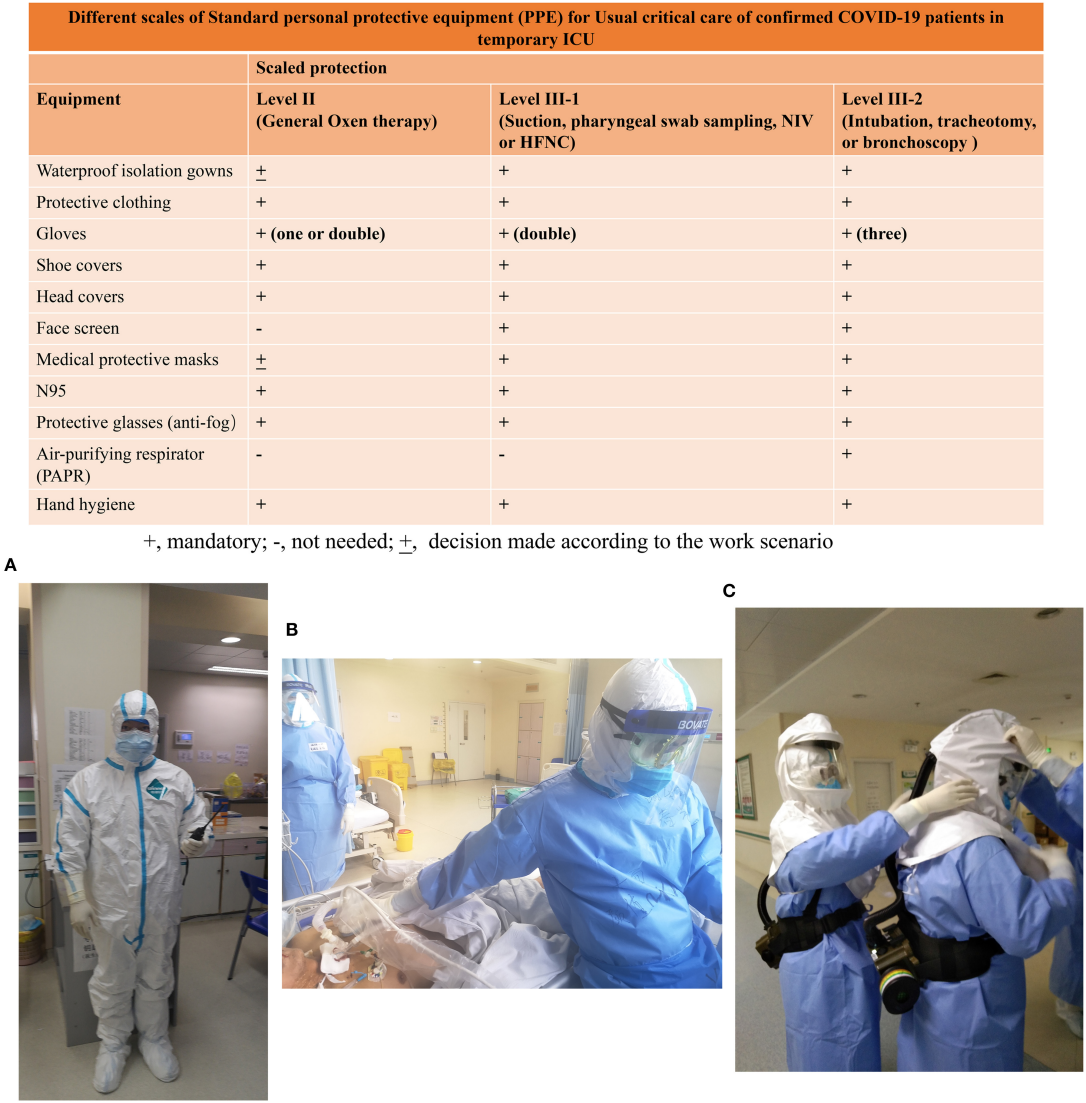
Standard personal protective equipment (PPE) required for normal critical care with COVID-confirmed patients in a temporary ICU. The required standard of PPE is based on the guidelines for the prevention and control of COVID-19 infection in medical institutions laid out by the government in China. Doctors have different scales of PPE to care for severe confirmed patients in different situations. **(A)** A doctor with level-II PPE at the nurses' station; **(B)** A doctor with level III-1 PPE for ultrasonography in a temporary ICU; **(C)** Two doctors with level III-2 PPE preparing for percutaneous tracheostomy in a temporary ICU.

Preventing a nosocomial outbreak of COVID-19 through transmission from patients to healthcare workers was of vital importance. To this end, every health worker had to be equipped with the correct personal protective equipment (PPE) and given training in its use before they could start caring for patients. After donning a medical protective mask, every worker was required to ask another to check if it was sufficiently tight, and, after they took their PPE off, they used quick-drying hand sanitizer. Different levels of PPE were assigned to healthcare workers according to the severity of the patients under their care, as showed in Figure 5. Workers administering oxygen therapy to confirmed COVID-19 patients in the third sector wore level-II protection consisting of protective clothing, gloves, shoe covers, head covers, a N95 mask, and protective (anti-fog) glasses, and they also carried out hand hygiene measures. Personnel carrying out operations among patients that might be associated with aerosol generating procedures, like sampling or sputum suction, wore level III-1 protection consisting of all the above items plus a waterproof isolation gown, and a face shield and also carried out the hand hygiene procedures. Personnel caring for sector-two patients, suffering from severe respiratory distress but not requiring invasive ventilation treatment, were also obliged to use level III-1 PPE (8). According to recent recommendations (10, 11), the highest level of PPE, level III-2, which included an air-purifying respirators (PAPRs), was reserved for high risk operations including tracheal intubation, tracheotomy, or fiberoptic bronchoscopy.

### Education and Training of Health Workers

It is estimated that a considerable proportion of healthcare workers have been infected since the COVID-19 outbreak (for example, infected rates of health workers were 28603/236076 (12.1%) in Italy (https://www.epicentro.iss.it/en/coronavirus/bollettino/Infografica_10giugno%20ENG.pdf). Minimizing the risk of nosocomial outbreak amplification and protecting healthcare workers are of critical importance (8, 12). Widespread use of recommended barrier precautions in isolated wards must be of highly priority. The temporary ICUs set up to manage the epidemic in China established protocols for both the prevention of infection and contingency management, including PPE regulations, procedures for entering and leaving quarantined zones, emergency plans to cope with occupational exposure, checks on the hygiene standards observed by healthcare workers, and management of preventive medication. It is imperative that ensuring every healthcare worker is properly trained, continually protected against droplet infection, and versed in all the necessary checks and precautions, including the use of PPE. This is vital in the battle against a highly infectious disease such as COVID-19.

## Management

Data from a follow-up study of 99 COVID-19 cases, dated January 25, 2020, reveal that 11% died, 9% required invasive mechanical ventilation, and 3% required extracorporeal membrane oxygenation (ECMO) (13). Among the significant findings was that before January 30, 2020, only about 25% of patients who died from COVID-19 received invasive mechanical ventilation or ECMO, and that HFNC and/or non-invasive ventilation (NIV) were used for an average of 6 (4–8) days before intubation or death (14). It can be inferred that invasive mechanical ventilation was delayed, possibly due to a lack of the necessary equipment, specialist staff, or areas in which the procedure could be carried out or to a fear of infection during the operation of trachea intubation.

COVID-19 is an infectious disease characterized as an acute hypoxemic respiratory insufficiency or failure which requires the use of oxygen and ventilation therapies. Most infected patients suffer mild symptoms and are self-healing; however, in severe cases, progression is rapid and can lead to ARDS, septic shock, metabolic acidosis, and coagulopathy (15), especially when combined with old age, comorbidities, or persistent lymphocytopenia, which is difficult to correct. As standardizing the management of care is a vital element in improving survival rates, the National Health Commission of the People's Republic of China (http://www.nhc.gov.cn/yzygj/s7653p/202002/d4b895337e19445f8d728fcaf1e3e13a.shtml) and the WHO (https://www.who.int/docs/default-source/coronaviruse/clinical-management-of-novel-cov.pdf) have established a protocol for the treatment of COVID-19. Timely and effective airway management and maximized first-pass success rate of airway operation for COVID-19 patients are recommended (8, 11).

For patients in the third sector of the temporary ICU (who have mild symptoms), healthcare workers must give supportive care and ensure monitoring at 6-h intervals of the vital symptoms, such as breathing rate, oxygen saturation (SpO2), and heart rate. Oxygen therapy should be initiated at 5 L/min and titrated to SpO2 ≥ 92% in COVID-19 patients. For patients with high risk factors (≥66 years old and comorbidities), nurses must ensure close monitoring of vital symptoms at 4-h intervals. In the following conditions, patients must be swiftly transferred to the second (moderate) sector, in case they need HFNC oxygenation or NIV: breathing rate ≥24/min, SpO2 <92% with oxygen therapy at 5 L/min, or heart rate >130/min, or persistent lymphocytopenia. Throughout treatment, conservative intravenous fluid strategies must be strictly implemented unless septic shock occurs.

**Figure 6:**
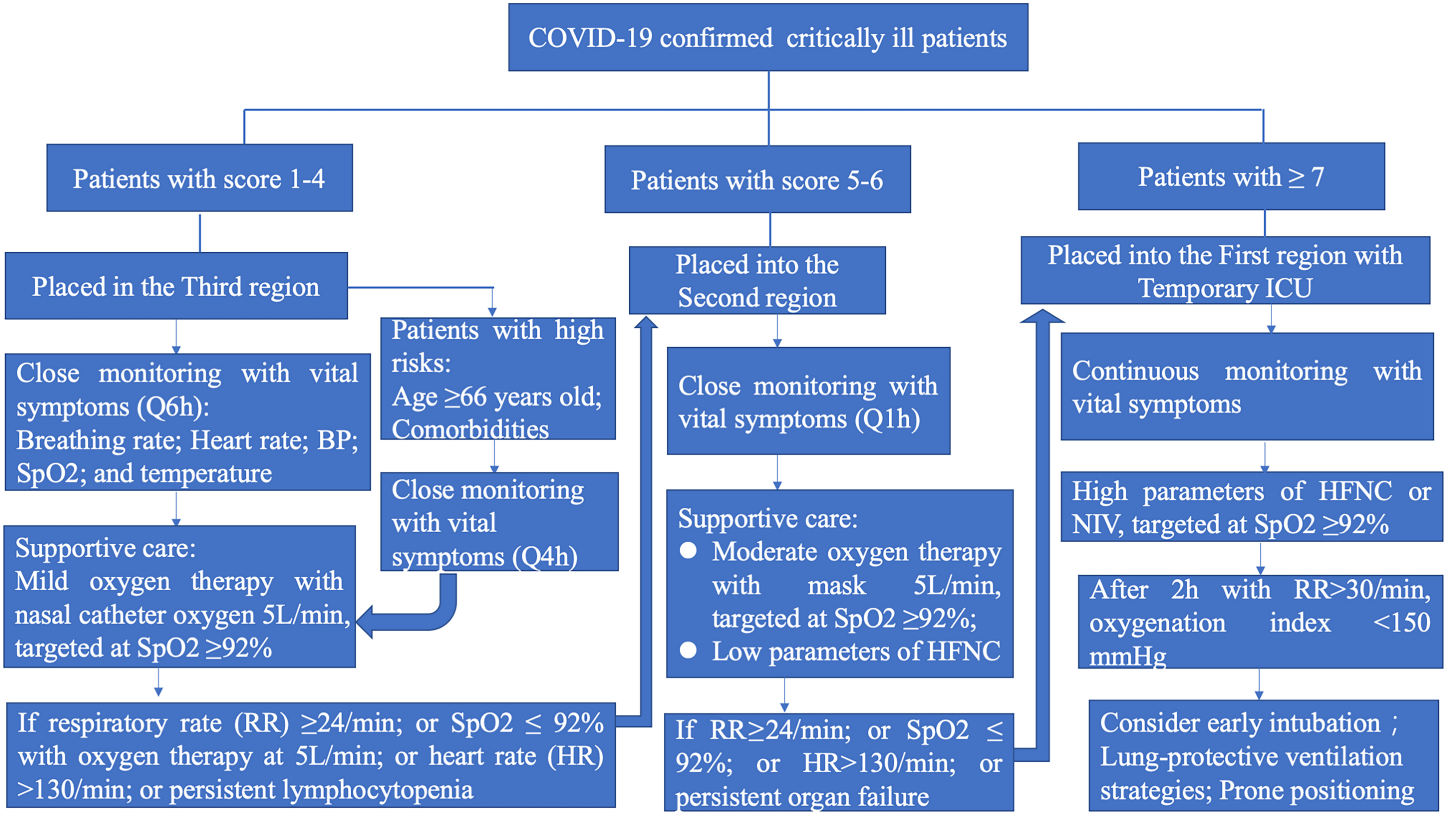
The management algorithm based on the clinical severity of critically ill COVID-19 patients. HFNC, high flow nasal cannula; NIV, non-invasive ventilation.

In the case of severe patients with persistent respiratory distress, a respiratory rate >30/min, oxygenation index <150 mmHg, or showing no improvement after HFNC or NIV, continuous monitoring is necessary from an experienced ICU team. The vital symptoms must be monitored after HFNC or NIV treatment at intervals of no more than 2 h (16). If there is no improvement after 2 h, or the patient's condition worsens, the team should consider early invasive ventilation (8), followed by lung-protective ventilation strategies and early prone positioning during mechanical ventilation for more than 12 hours per day (Figure 6).

## Conclusions

The sudden outbreak of the highly contagious disease now known as COVID-19 in China in December 2019 has impacted healthcare system and societies more widely across the world. Should another virus of this type emerge, it will be vital to first control the source of infection and second prevent transmission. Moreover, the identification, sorting, and management of infected patients in different isolated sectors according to level of severity is crucial in ensuring full use is made of the medical resources available. Public anxiety around COVID-19 centers on the large numbers of critically ill patients and high death rates. In this regard, setting up a temporary ICU in isolated hospitals, run by multidisciplinary staff under the leadership of intensive care specialists and nurses, is crucial not only in caring for critically ill patients and bringing down mortality rates, but also in allaying public fear.

## Data Availability Statement

The original contributions presented in the study are included in the article/supplementary material, further inquiries can be directed to the corresponding author/s.

## Author Contributions

MP contributed to the literature search, figures, writing of the original draft, and project administration of the manuscript. ZQ and LZ contributed equally to the conceptualization, data and resource curation, supervision, validation, and writing, reviewing, and editing of the research. All authors contributed to the article and approved the submitted version.

## Conflict of Interest

The authors declare that the research was conducted in the absence of any commercial or financial relationships that could be construed as a potential conflict of interest.

## References

[1] Wu Z McGoogan JM. Characteristics of and important lessons from the coronavirus disease 2019 (COVID-19) outbreak in China: summary of a report of 72314 cases from the Chinese Center for Disease Control and Prevention. JAMA. (2020) 323:1239–42. 10.1001/jama.2020.2648 32091533

[2] Yang X Yu Y Xu J Shu H Xia J Liu H. Clinical course and outcomes of critically ill patients with SARS-CoV-2 pneumonia in Wuhan, China: a single-centered, retrospective, observational study. Lancet Respir Med. (2020) 8:475–81. 10.1016/S2213-2600(20)30079-5 32105632 PMC7102538

[3] Murthy S Gomersall CD Fowler RA. Care for critically ill patients with COVID-19. JAMA. (2020) 323:1499–500. 10.1001/jama.2020.3633 32159735

[4] Maves RC Jamros CM Smith AG. Intensive care unit preparedness during pandemics and other biological threats. Crit Care Clin. (2019) 35:609–18. 10.1016/j.ccc.2019.06.001 31445608 PMC7134984

[5] Liao X Wang B Kang Y. Novel coronavirus infection during the 2019-2020 epidemic: preparing intensive care units-the experience in Sichuan Province, China. Intensive Care Med. (2020) 46:357–60. 10.1007/s00134-020-05954-2 32025779 PMC7042184

[6] Smith ME Chiovaro JC O'Neil M Kansagara D Quinones AR Freeman M. Early warning system scores for clinical deterioration in hospitalized patients: a systematic review. Ann Am Thorac Soc. (2014) 11:1454–65. 10.1513/AnnalsATS.201403-102OC 25296111

[7] Wang D Hu B Hu C Zhu F Liu X Zhang J. Clinical characteristics of 138 hospitalized patients with 2019 Novel Coronavirus-Infected Pneumonia in Wuhan, China. JAMA. (2020) 323:1061–69. 10.1001/jama.2020.1585 32031570 PMC7042881

[8] Sorbello M El-Boghdadly K Di Giacinto I Cataldo R Esposito C Falcetta S. The Italian coronavirus disease 2019 outbreak: recommendations from clinical practice. Anaesthesia. (2020) 75:724–32. 10.1111/anae.15049 32221973

[9] Ruan Q Yang K Wang W Jiang L Song J Clinical predictors of mortality due to COVID-19 based on an analysis of data of 150 patients from Wuhan, China. Intensive Care Med. (2020) 46:846–8. 10.1007/s00134-020-05991-x 32125452 PMC7080116

[10] Orser BA. Recommendations for Endotracheal Intubation of COVID-19 Patients. Anesth Analg. (2020) 130: 1109–10. 10.1213/ANE.0000000000004803 32209810 PMC7172572

[11] Cook TM El-Boghdadly K McGuire B McNarry AF Patel A Higgs A. Consensus guidelines for managing the airway in patients with COVID-19: guidelines from the Difficult Airway Society, the Association of Anaesthetists the Intensive Care Society, the Faculty of Intensive Care Medicine and the Royal College of Anaesthetists. Anaesthesia. (2020) 75:785–99. 10.1111/anae.15054 32221970 PMC7383579

[12] Adams JG Walls RM. Supporting the health care workforce during the COVID-19 global epidemic. JAMA. (2020). 10.1001/jama.2020.3972 32163102

[13] Chen N Zhou M Dong X Qu J Gong F Han Y. Epidemiological and clinical characteristics of 99 cases of 2019 novel coronavirus pneumonia in Wuhan, China: a descriptive study. Lancet. (2020) 395:507–13. 10.1016/S0140-6736(20)30211-7 32007143 PMC7135076

[14] Xie J Tong Z Guan X Du B Qiu H Slutsky AS. Critical care crisis and some recommendations during the COVID-19 epidemic in China. Intensive Care Med. (2020) 46:837–40. 10.1007/s00134-020-05979-7 32123994 PMC7080165

[15] Robba C Battaglini D Ball L Patroniti N Loconte M Brunetti I. Distinct phenotypes require distinct respiratory management strategies in severe COVID-19. Respir Physiol Neurobiol. (2020) 279:103455. 10.1016/j.resp.2020.103455 32437877 PMC7211757

[16] Zuo MZ Huang YG Ma WH Xue ZG Zhang JQ Gong YH. Expert recommendations for tracheal intubation in critically ill patients with Noval Coronavirus Disease 2019. Chin Med Sci J. (2020) 35:105–9. 10.24920/003724 32102726 PMC7367670

